# Oscillation of a Class of Fractional Differential Equations with Damping Term

**DOI:** 10.1155/2013/685621

**Published:** 2013-08-21

**Authors:** Huizeng Qin, Bin Zheng

**Affiliations:** School of Science, Shandong University of Technology, Zibo, Shandong 255049, China

## Abstract

We investigate the oscillation of a class of fractional differential equations with damping term. Based on a certain variable transformation, the fractional differential equations are converted into another differential equations of integer order with respect to the new variable. Then, using Riccati transformation, inequality, and integration average technique, some new oscillatory criteria for the equations are established. As for applications, oscillation for two certain fractional differential equations with damping term is investigated by the use of the presented results.

## 1. Introduction

 In the investigations of qualitative properties for differential equations, research of oscillation has gained much attention by many authors in the last few decades (e.g., see [1–16]). In these investigations, we notice that very little attention is paid to oscillation of fractional differential equations.

In [17], Jumarie proposed a definition for fractional derivative which is known as the modified Riemann-Liouville derivative in the literature. Since then, many authors have investigated various applications of the modified Riemann-Liouville derivative (e.g., see [18–21]) including various fractional calculus formulae, the fractional variational iteration method, the Bäcklund transformation method, and the fractional subequation method for soling fractional partial differential equations. In this paper, based on the modified Riemann-Liouville derivative, we are concerned with oscillation of a class of fractional differential equations with damping term as follows:

(1) Dtα[a(t)Dtα(r(t)Dtαx(t))]+p(t)Dtα(r(t)Dtαx(t))+q(t)x(t)=0, t≥t0>0,  0<α<1,

where *D*_*t*_^*α*^(·) denotes the modified Riemann-Liouville derivative with respect to the variable *t*, the function *a* ∈ *C* ^*α*^([*t*_0_, *∞*), *R*_+_), *r* ∈ *C* ^2*α*^([*t*_0_, *∞*), *R*_+_), *p*, *q* ∈ *C*([*t*_0_, *∞*), *R*_+_) and *C* ^*α*^ denotes continuous derivative of order *α*.

The definition and some important properties for the modified Riemann-Liouville derivative of order *α* are listed as follows (see also in [20–24]):

(2) Dtαf(t) ={1Γ(1−α)ddt×∫0t(t−ξ)−α(f(ξ)−f(0))dξ,0<α<1,(f(n)(t))(α−n),n≤α<n+1,  n≥1,

(3) $$\begin{array}{c} D_{t}^{\alpha }t^{r}=\frac{\Gamma \left(1+r\right)}{\Gamma \left(1+r-\alpha \right)}t^{r-\alpha }, \end{array}$$

(4) $$\begin{array}{c} D_{t}^{\alpha }\left(f\left(t\right)g\left(t\right)\right)=g\left(t\right)D_{t}^{\alpha }f\left(t\right)+f\left(t\right)D_{t}^{\alpha }g\left(t\right), \end{array}$$

(5) $$\begin{array}{c} D_{t}^{\alpha }f\left[g\left(t\right)\right]=f_{g}^{\prime }\left[g\left(t\right)\right]D_{t}^{\alpha }g\left(t\right)=D_{g}^{\alpha }f\left[g\left(t\right)\right]{\left(g^{\prime }\left(t\right)\right)}^{\alpha }. \end{array}$$

As usual, a solution *x*(*t*) of (1) is called oscillatory if it has arbitrarily large zeros; otherwise, it is called nonoscillatory. Equation (1) is called oscillatory if all its solutions are oscillatory.

We organize the next as follows. In Section 2, using Riccati transformation, inequality, and integration average technique, we establish some new oscillatory criteria for (1), while we present some examples for them in Section 3.

## 2. Oscillatory Criteria for (1)

 In the following, we denote *ξ* = *t* ^*α*^/Γ(1 + *α*), *ξ*_*i*_ = *t*_*i*_^*α*^/Γ(1 + *α*), *i* = 0,1, 2,3, 4,5, $a(t)=\tilde{a}(\xi )$, $r(t)=\tilde{r}(\xi )$, $p(t)=\tilde{p}(\xi )$, $q(t)=\tilde{q}(\xi )$, *R*_+_ = (0, *∞*), ${\tilde{\delta }}_{1}(\xi ,\xi_{i})=\int_{\xi_{i}}^{\xi }\left(1/{\tilde{a}}^{1/\gamma }(s)\right)ds$, $\delta_{1}(t,t_{i})={\tilde{\delta }}_{1}(\xi ,\xi_{i})$, ${\tilde{\delta }}_{2}(\xi ,\xi_{i})=\int_{\xi_{i}}^{\xi }\left({\tilde{\delta }}_{1}(s,\xi_{i})/\tilde{r}(s)\right)ds$, $\delta_{2}(t,t_{i})={\tilde{\delta }}_{2}(\xi ,\xi_{i})$, $A(\xi )=\exp(\int_{\xi_{0}}^{\xi }\left(\tilde{p}(s)/\tilde{a}(s)\right)ds)$. Let *h*_1_, *h*_2_, *H* ∈ *C*([*ξ*_0_, *∞*), *R*) satisfy

(6) $$\begin{array}{c} H\left(\xi ,\xi \right)=0,\;H\left(\xi ,s\right)>0,\;\xi >s\geq \xi_{0}. \end{array}$$

*H* has continuous partial derivatives ∂*H*(*ξ*, *s*)/∂*ξ* and ∂*H*(*ξ*, *s*)/∂*s* on [*ξ*_0_, *∞*) such that

(7) $$\begin{array}{c} \frac{\partial H\left(\xi ,s\right)}{\partial \xi }=-h_{1}\left(\xi ,s\right)\sqrt{H\left(\xi ,s\right)},\\ \frac{\partial H\left(\xi ,s\right)}{\partial s}=-h_{2}\left(\xi ,s\right)\sqrt{H\left(\xi ,s\right)},\;\;\xi >s\geq \xi_{0}. \end{array}$$

**Lemma 1**

Assume *x*(*t*) is an eventually positive solution of (1), and

(8) $$\begin{array}{c} \int_{\xi_{0}}^{\infty }\frac{1}{A\left(s\right)\tilde{a}\left(s\right)}ds=\infty , \end{array}$$

(9) $$\begin{array}{c} \int_{t_{0}}^{\infty }\frac{\alpha t^{\alpha -1}}{\Gamma \left(1+\alpha \right)r\left(t\right)}dt=\infty , \end{array}$$

(10) $$\begin{array}{c} \int_{\xi_{0}}^{\infty }\frac{1}{\tilde{r}\left(\zeta \right)}\int_{\zeta }^{\infty }\frac{1}{A\left(\tau \right)\tilde{a}\left(\tau \right)}\int_{\tau }^{\infty }A\left(s\right)\tilde{q}\left(s\right)ds\;d\tau \;d\zeta =\infty . \end{array}$$

Then, there exists a sufficiently large *T* such that *D*_*t*_^*α*^(*r*(*t*)*D*_*t*_^*α*^ *x*(*t*)) > 0 on [*T*, *∞*) and either *D*_*t*_^*α*^ *x*(*t*) > 0 on [*T*, *∞*) or lim⁡_*t*→*∞*_ *x*(*t*) = 0.

**Proof**

Let $x(t)=\tilde{x}(\xi )$, where *ξ* = *t* ^*α*^/Γ(1 + *α*). Then, by use of (3), we obtain *D*_*t*_^*α*^ *ξ*(*t*) = 1, and furthermore, by use of the first equality in (5), we have

(11) $$\begin{array}{c} D_{t}^{\alpha }x\left(t\right)=D_{t}^{\alpha }\tilde{x}\left(\xi \right)={\tilde{x}}^{\prime }\left(\xi \right)D_{t}^{\alpha }\xi \left(t\right)={\tilde{x}}^{\prime }\left(\xi \right). \end{array}$$

Similarly, we have $D_{t}^{\alpha }a\left(t\right)={\tilde{a}}^{\prime }\left(\xi \right)$, $D_{t}^{\alpha }r(t)={\tilde{r}}^{\prime }(\xi )$. So, (1) can be transformed into the following form:

(12) [a~(ξ)(r~(ξ)x~′(ξ))′]′+p~(ξ)(r~(ξ)x~′(ξ))′+q~(ξ)x~(ξ)=0, ξ≥ξ0>0.

Since *x*(*t*) is an eventually positive solution of (1), then $\tilde{x}(\xi )$ is an eventually positive solution of (12), and there exists *ξ*_1_ > *ξ*_0_ such that $\tilde{x}(\xi )>0$ on [*ξ*_1_, *∞*). Furthermore, we have

(13) [A(ξ)a~(ξ)(r~(ξ)x~′(ξ))′]′=A(ξ)[a~(ξ)(r~(ξ)x~′(ξ))′]′+A′(ξ)a~(ξ)(r~(ξ)x~′(ξ))′=A(ξ)[a~(ξ)(r~(ξ)x~′(ξ))′]′+A(ξ)p~(ξ)((r~(ξ)x~′(ξ))′)γ=−A(ξ)q~(ξ)x~(ξ)<0, ξ≥ξ1.

Then, $A\left(\xi \right)\tilde{a}\left(\xi \right){\left(\tilde{r}\left(\xi \right){\tilde{x}}^{\prime }\left(\xi \right)\right)}^{\prime }$ is strictly decreasing on [*ξ*_1_, *∞*), and thus ${\left(\tilde{r}\left(\xi \right){\tilde{x}}^{\prime }\left(\xi \right)\right)}^{\prime }$ is eventually of one sign. We claim ${\left(\tilde{r}\left(\xi \right){\tilde{x}}^{\prime }\left(\xi \right)\right)}^{\prime }>0$ on [*ξ*_2_, *∞*), where *ξ*_2_ > *ξ*_1_ is sufficiently large. Otherwise, assume that there exists a sufficiently large *ξ*_3_ > *ξ*_2_ such that ${\left(\tilde{r}\left(\xi \right){\tilde{x}}^{\prime }\left(\xi \right)\right)}^{\prime }<0$ on [*ξ*_3_, *∞*). Then, $\tilde{r}\left(\xi \right){\tilde{x}}^{\prime }(\xi )$ is strictly decreasing on [*ξ*_3_, *∞*), and we have

(14) r~(ξ)x~′(ξ)−r~(ξ3)x~′(ξ3)=∫ξ3ξA(s)a~(s)(r~(s)x~′(s))′A(s)a~(s)ds≤A(ξ3)a~(ξ3)(r~(ξ3)x~′(ξ3))′×∫ξ3ξ1A(s)a~(s)ds.

By (8), we have $\lim_{\xi \to \infty }\tilde{r}\left(\xi \right){\tilde{x}}^{\prime }(\xi )=-\infty$. So there exists a sufficiently large *ξ*_4_ with *ξ*_4_ > *ξ*_3_ such that ${\tilde{x}}^{\prime }(\xi )<0$, *ξ* ∈ [*ξ*_4_, *∞*). Furthermore,

(15) x~(ξ)−x~(ξ4)=∫ξ4ξx~′(s)ds=∫ξ4ξr~(s)x~′(s)r~(s)ds≤r~(ξ4)x~′(ξ4)∫ξ4ξ1r~(s)ds=r~(ξ4)x~′(ξ4)∫t4∞αtα−1Γ(1+α)r(t)dt.

By (9), we deduce that $\lim_{\xi \to \infty }\tilde{x}(\xi )=-\infty$, which contradicts the fact that $\tilde{x}(\xi )$ is an eventually positive solution of (9). So, ${\left(\tilde{r}\left(\xi \right){\tilde{x}}^{\prime }\left(\xi \right)\right)}^{\prime }>0$ on [*ξ*_2_, *∞*), and *D*_*t*_^*α*^(*r*(*t*)*D*_*t*_^*α*^ *x*(*t*)) > 0 on [*t*_2_, *∞*). Thus, $D_{t}^{\alpha }x(t)={\tilde{x}}^{\prime }(\xi )$ is eventually of one sign. Now we assume ${\tilde{x}}^{\prime }(\xi )<0$, *ξ* ∈ [*ξ*_5_, *∞*) for some sufficiently large *ξ*_5_ > *ξ*_4_. Since $\tilde{x}(\xi )>0$, furthermore we have $\lim_{\xi \to \infty }\tilde{x}(\xi )=\beta \geq 0$. We claim *β* = 0. Otherwise, assume *β* > 0. Then $\tilde{x}(\xi )\geq \beta$ on [*ξ*_5_, *∞*), and, for *ξ* ∈ [*ξ*_5_, *∞*), by (12) we have

(16) [A(ξ)a~(ξ)(r~(ξ)x~′(ξ))′]′≤−A(ξ)q~(ξ)x~(ξ)≤−A(ξ)q~(ξ)β.

Substituting *ξ* with *s* in the previous inequality, an integration with respect to *s* from *ξ* to *∞* yields

(17) −A(ξ)a~(ξ)(r~(ξ)x~′(ξ))′≤−lim⁡ξ→∞A(ξ)a~(ξ)(r~(ξ)x~′(ξ))′ −β∫ξ∞A(s)q~(s)ds<−β∫ξ∞A(s)q~(s)ds,

which means

(18) $$\begin{array}{c} {\left(\tilde{r}\left(\xi \right){\tilde{x}}^{\prime }\left(\xi \right)\right)}^{\prime }>\frac{\beta }{A\left(\xi \right)\tilde{a}\left(\xi \right)}\int_{\xi }^{\infty }A\left(s\right)\tilde{q}\left(s\right)ds. \end{array}$$

Substituting *ξ* with *τ* in (18), an integration for (18) with respect to *τ* from *ξ* to *∞* yields

(19) −r~(ξ)x~′(ξ)>−lim⁡ξ→∞r~(ξ)x~′(ξ)+β∫ξ∞1A(τ)a~(τ)∫τ∞A(s)q~(s)ds dτ>β∫ξ∞1A(τ)a~(τ)∫τ∞A(s)q~(s)ds dτ;

that is,

(20) $$\begin{array}{c} {\tilde{x}}^{\prime }\left(\xi \right)<-\beta \frac{1}{\tilde{r}\left(\xi \right)}\int_{\xi }^{\infty }\frac{1}{A\left(\tau \right)\tilde{a}\left(\tau \right)}\int_{\tau }^{\infty }A\left(s\right)\tilde{q}\left(s\right)ds\;d\tau . \end{array}$$

Substituting *ξ* with *ζ* in (20), an integration for (20) with respect to *ζ* from *ξ*_5_ to *ξ* yields

(21) x~(ξ)−x~(ξ5)<−β∫ξ5ξ1r~(ζ)∫ζ∞1A(τ)a~(τ)∫τ∞A(s)q~(s)ds dτ dζ.

By (10), one can see $\lim_{t\to \infty }\tilde{x}(\xi )=-\infty$, which causes a contradiction. So, the proof is complete.

**Lemma 2**

Assume that *x* is an eventually positive solution of (1) such that

(22) $$\begin{array}{c} D_{t}^{\alpha }\left(r\left(t\right)D_{t}^{\alpha }x\left(t\right)\right)>0,\;\;D_{t}^{\alpha }x\left(t\right)>0 \end{array}$$

on [*t*_1_, *∞*)_*𝕋*_, where *t*_1_ ≥ *t*_0_ is sufficiently large. Then, for *t* ≥ *t*_1_, we have

(23) $$\begin{array}{c} D_{t}^{\alpha }x\left(t\right)\geq \frac{A\left(\xi \right)\delta_{1}\left(t,t_{1}\right)a\left(t\right)D_{t}^{\alpha }\left(r\left(t\right)D_{t}^{\alpha }x\left(t\right)\right)}{r\left(t\right)}, \end{array}$$

(24) $$\begin{array}{c} x\left(t\right)\geq A\left(\xi \right)\delta_{2}\left(t,t_{1}\right)a\left(t\right)D_{t}^{\alpha }\left(r\left(t\right)D_{t}^{\alpha }x\left(t\right)\right). \end{array}$$

**Proof**

By (13), we obtain that $A\left(\xi \right)\tilde{a}\left(\xi \right){\left(\tilde{r}\left(\xi \right){\tilde{x}}^{\prime }\left(\xi \right)\right)}^{\prime }$ is strictly decreasing on [*ξ*_1_, *∞*). So,

(25) r~(ξ)x~′(ξ)≥r~(ξ)x~′(ξ)−r~(ξ1)x~′(ξ1)=∫ξ1ξA(s)a~(s)(r~(s)x~′(s))′A(s)a~(s)ds≥A(ξ)a~(ξ)(r~(ξ)x~′(ξ))′∫ξ1ξ1A(s)a~(s)ds=A(ξ)a~(ξ)(r~(ξ)x~′(ξ))′δ~1(ξ,ξ1);

that is

(26) $$\begin{array}{c} r\left(t\right)D_{t}^{\alpha }x\left(t\right)\geq A\left(\xi \right)\delta_{1}\left(t,t_{1}\right)a\left(t\right)D_{t}^{\alpha }\left(r\left(t\right)D_{t}^{\alpha }x\left(t\right)\right), \end{array}$$

which admits (23). On the other hand, we have

(27) x~(ξ)≥x~(ξ)−x~(ξ1)≥∫ξ1ξA(s)δ~1(s,ξ1)a~(s)(r~(s)x~′(s))′A(s)r~(s)ds≥A(ξ)a~(ξ)(r~(ξ)x~′(ξ))′∫ξ1ξδ~1(s,ξ1)A(s)r~(s)ds=A(ξ)δ~2(ξ,ξ1)a~(ξ)(r~(ξ)x~′(ξ))′,

which can be rewritten as (24). So the proof is complete.

**Lemma 3 (see [25, Theorem 41])**

Assume that *A* and *B* are nonnegative real numbers. Then,

(28) $$\begin{array}{c} \lambda AB^{\lambda -1}-A^{\lambda }\leq \left(\lambda -1\right)B^{\lambda } \end{array}$$

for all *λ* > 1.

**Theorem 4**

Assume that (8)–(10) hold. If there exists *ϕ* ∈ *C* ^*α*^([*t*_0_, *∞*), *R*_+_) such that for any sufficiently large *T* ≥ *ξ*_0_, there exist *a*, *b*, *c* with *T* ≤ *a* < *c* < *b* satisfying

(29) 1H(b,c)∫cbH(b,s)A(s)ϕ~(s)q~(s)ds+1H(c,a)∫acH(s,a)A(s)ϕ~(s)q~(s)ds>1H(b,c)∫cbr~(s)ϕ~(s)4δ~1(s,ξ2)Q22(b,s)ds+1H(c,a)∫acr~(s)ϕ~(s)4δ~1(s,ξ2)Q12(s,a)ds,

where $\tilde{\phi }(\xi )=\phi (t)$, $Q_{1}(s,\xi )=h_{1}(s,\xi )-\left({\tilde{\phi }}^{\prime }(s)/\tilde{\phi }(s)\right)\sqrt{H(s,\xi )}$,  $Q_{2}(\xi ,s)=h_{2}(\xi ,s)-\left({\tilde{\phi }}^{\prime }(s)/\tilde{\phi }(s)\right)\sqrt{H(\xi ,s)}$; then, (1) is oscillatory or satisfies lim⁡_*t*→*∞*_ *x*(*t*) = 0.

**Proof**

Assume that (1) has a nonoscillatory solution *x* on [*t*_0_, *∞*). Without loss of generality, we may assume that *x*(*t*) > 0 on [*t*_1_, *∞*), where *t*_1_ is sufficiently large. By Lemma 1, we have *D*_*t*_^*α*^(*r*(*t*)*D*_*t*_^*α*^ *x*(*t*)) > 0, *t* ∈ [*t*_2_, *∞*), where *t*_2_ > *t*_1_ is sufficiently large, and either *D*_*t*_^*α*^ *x*(*t*) > 0 on [*t*_2_, *∞*) or lim⁡_*t*→*∞*_ *x*(*t*) = 0. Now we assume *D*_*t*_^*α*^ *x*(*t*) > 0 on [*t*_2_, *∞*). Define the generalized Riccati function:

(30) $$\begin{array}{c} \omega \left(t\right)=\phi \left(t\right)\frac{A\left(\xi \right)a\left(t\right)D_{t}^{\alpha }\left(r\left(t\right)D_{t}^{\alpha }x\left(t\right)\right)}{x\left(t\right)}. \end{array}$$

Then, for *t* ∈ [*t*_2_, *∞*), we have

(31) Dtαω(t)=Dtαϕ(t)A(ξ)a(t)Dtα(r(t)Dtαx(t))x(t)+ϕ(t)Dtα{A(ξ)a(t)Dtα(r(t)Dtαx(t))x(t)}=ϕ(t)Dtα(A(ξ)a(t)Dtα(r(t)Dtαx(t)))x(t)−ϕ(t)Dtαx(t)A(ξ)a(t)Dtα(r(t)Dtαx(t))x2(t)+Dtαϕ(t)ϕ(t)ω(t)=(ϕ(t)[A(ξ)Dtα(a(t)Dtα(r(t)Dtαx(t)))+a(t)Dtα(r(t)Dtαx(t))DtαA(ξ)])×(x(t))−1−ϕ(t)Dtαx(t)A(ξ)a(t)Dtα(r(t)Dtαx(t))x2(t)+Dtαϕ(t)ϕ(t)ω(t)=(ϕ(t)[A(ξ)Dtα(a(t)Dtα(r(t)Dtαx(t)))+a(t)Dtα(r(t)Dtαx(t))A′(ξ)Dtαξ])×(x(t))−1−ϕ(t)Dtαx(t)A(ξ)a(t)Dtα(r(t)Dtαx(t))x2(t)+Dtαϕ(t)ϕ(t)ω(t).

Using *D*_*t*_^*α*^ *ξ* = 1 and (23), we obtain

(32) Dtαω(t)≤(ϕ(t)[A(ξ)Dtα(a(t)Dtα(r(t)Dtαx(t)))+a(t)Dtα(r(t)Dtαx(t))A(ξ)p~(ξ)a~(ξ)])×(x(t))−1−ϕ(t)((A(ξ)δ1(t,t2)a(t)Dtα(r(t)Dtαx(t))×Dtαx(t)A(ξ)a(t)Dtα(r(t)Dtαx(t)))×(r(t)x2(t))−1)+Dtαϕ(t)ϕ(t)ω(t)=(ϕ(t)A(ξ)[Dtα(a(t)Dtα(r(t)Dtαx(t)))+p(t)Dtα(r(t)Dtαx(t))])×(x(t))−1−δ1(t,t2)ϕ(t)r(t)ω2(t)+Dtαϕ(t)ϕ(t)ω(t)=−A(ξ)q(t)ϕ(t)−δ1(t,t2)ϕ(t)r(t)ω2(t)+Dtαϕ(t)ϕ(t)ω(t).

Let $\omega (t)=\tilde{\omega }(\xi )$. Then $D_{t}^{\alpha }w(t)={\tilde{w}}^{\prime }(\xi )$, and $D_{t}^{\alpha }\phi (t)={\tilde{\phi }}^{\prime }(\xi )$. So (32) is transformed into the following form:

(33) ω~′(ξ)≤−A(ξ)ϕ~(ξ)q~(ξ)−δ~1(ξ,ξ2)r~(ξ)ϕ~(ξ)ω~2(ξ) +ϕ~′(ξ)ϕ~(ξ)ω~(ξ), ξ≥ξ2.

Choose *a*, *b*, *c* arbitrarily in [*ξ*_2_, *∞*) with *b* > *c* > *a*. Substituting *ξ* with *s*, multiplying both sides of (33) by *H*(*ξ*, *s*), and integrating it with respect to *s* from *c* to *ξ* for *ξ* ∈ [*c*, *b*), we get that

(34) ∫cξH(ξ,s)A(s)ϕ~(s)q~(s)ds≤−∫cξH(ξ,s)w~′(s)ds+∫cξH(ξ,s)ϕ~′(s)ϕ~(s)w~(s)ds−∫cξH(ξ,s)δ~1(s,ξ2)r~(s)ϕ~(s)ω~2(s)ds=H(ξ,c)w~(c)−∫cξ[(δ~1(s,ξ2)H(ξ,s)r~(s)ϕ~(s))1/2w~(s)+12(r~(s)ϕ~(s)δ~1(s,ξ2))1/2Q2(ξ,s)]2ds+∫cξr~(s)ϕ~(s)4δ~1(s,ξ2)Q22(ξ,s)ds≤H(ξ,c)w~(c)+∫cξr~(s)ϕ~(s)4δ~1(s,ξ2)Q22(ξ,s)ds.

Dividing both sides of the inequality (34) by *H*(*ξ*, *c*) and letting *ξ* → *b* ^−^, we obtain

(35) 1H(b,c)∫cbH(b,s)A(s)ϕ~(s)q~(s)ds≤w~(c)+1H(b,c)∫cbr~(s)ϕ~(s)4δ~1(s,ξ2)Q22(b,s)ds.

On the other hand, substituting *ξ* with *s*, multiplying both sides of (33) by *H*(*s*, *ξ*), and integrating it with respect to *s* from *ξ* to *c* for *ξ* ∈ (*a*, *c*], we get that

(36) ∫ξcH(s,ξ)A(s)ϕ~(s)q~(s)ds≤−∫ξcH(s,ξ)w~′(s)ds+∫ξcH(s,ξ)ϕ~′(s)ϕ~(s)w~(s)ds−∫ξcH(s,ξ)δ~1(s,ξ2)r~(s)ϕ~(s)ω~2(s)ds=−H(c,ξ)w~(c)−∫ξc[(δ~1(s,ξ2)H(s,ξ)r~(s)ϕ~(s))1/2w~(s)+12(r~(s)ϕ~(s)δ~1(s,ξ2))1/2Q1(ξ,s)]2ds+∫ξcr~(s)ϕ~(s)4δ~1(s,ξ2)Q12(s,ξ)ds≤−H(c,ξ)w~(c)+∫ξcr~(s)ϕ~(s)4δ~1(s,ξ2)Q12(s,ξ)ds.

Dividing both sides of the inequality (36) by *H*(*c*, *ξ*) and letting *ξ* → *a* ^+^, we obtain

(37) 1H(c,a)∫acH(s,a)A(s)ϕ~(s)q~(s)ds≤−w~(c)+1H(c,a)∫acr~(s)ϕ~(s)4δ~1(s,ξ2)Q12(s,a)ds.

A combination of (35) and (37) yields

(38) 1H(b,c)∫cbH(b,s)A(s)ϕ~(s)q~(s)ds+1H(c,a)∫acH(s,a)A(s)ϕ~(s)q~(s)ds≤1H(b,c)∫cbr~(s)ϕ~(s)4δ~1(s,ξ2)Q22(b,s)ds+1H(c,a)∫acr~(s)ϕ~(s)4δ~1(s,ξ2)Q12(s,a)ds,

which contradicts (29). So, the proof is complete.

**Theorem 5**

Under the conditions of Theorem 4, if for any sufficiently large *l* ≥ *ξ*_0_,

(39) lim⁡ξ→∞sup⁡∫lξ[H(s,l)A(s)ϕ~(s)q~(s)−r~(s)ϕ~(s)4δ~1(s,ξ2)Q12(s,l)]ds>0,

(40) lim⁡ξ→∞sup⁡∫lξ[H(ξ,s)A(s)ϕ~(s)q~(s)−r~(s)ϕ~(s)4δ~1(s,ξ2)Q22(ξ,s)]ds>0,

then (1) is oscillatory.

**Proof**

For any *T* ≥ *ξ*_0_, let *a* = *T*. In (39), we choose *l* = *a*. Then, there exists *c* > *a* such that

(41) $$\begin{array}{c} \int_{a}^{c}\left[H\left(s,a\right)A\left(s\right)\tilde{\phi }\left(s\right)\tilde{q}\left(s\right)-\frac{\tilde{r}\left(s\right)\tilde{\phi }\left(s\right)}{4{\tilde{\delta }}_{1}\left(s,\xi_{2}\right)}Q_{1}^{2}\left(s,a\right)\right]ds>0. \end{array}$$

In (40), we choose *l* = *c* > *a*. Then there exists *b* > *c* such that

(42) $$\begin{array}{c} \int_{c}^{b}\left[H\left(b,s\right)A\left(s\right)\tilde{\phi }\left(s\right)\tilde{q}\left(s\right)-\frac{\tilde{r}\left(s\right)\tilde{\phi }\left(s\right)}{4{\tilde{\delta }}_{1}\left(s,\xi_{2}\right)}Q_{2}^{2}\left(b,s\right)\right]ds>0. \end{array}$$

Combining (41) and (42), we obtain (29). The conclusion thus comes from Theorem 4, and the proof is complete.

In Theorems 4 and 5, if we choose *H*(*ξ*, *s*) = (*ξ* − *s*)^*λ*^, *ξ* ≥ *s* ≥ *ξ*_0_, where *λ* > 1 is a constant, then we obtain the following two corollaries.

**Corollary 6**

Under the conditions of Theorem 4, if for any sufficiently large *T* ≥ *ξ*_0_, there exist *a*, *b*, *c* with *T* ≤ *a* < *c* < *b* satisfying

(43) 1(c−a)λ∫ac(s−a)λA(s)ϕ~(s)q~(s)ds+1(b−c)λ∫cb(b−s)λA(s)ϕ~(s)q~(s)ds>1(c−a)λ∫acr~(s)ϕ~(s)4δ~1(s,ξ2)(s−a)λ−2(λ+ϕ~′(s)ϕ~(s)(s−a))2ds+1(b−c)λ∫cbr~(s)ϕ~(s)4δ~1(s,ξ2)(b−s)λ−2×(λ−ϕ~′(s)ϕ~(s)(b−s))2ds,

then (1) is oscillatory.

**Corollary 7**

Under the conditions of Theorem 5, if for any sufficiently large *l* ≥ *ξ*_0_,

(44) lim⁡ξ→∞sup⁡∫lξ[(s−l)λA(s)ϕ~(s)q~(s)−r~(s)ϕ~(s)4δ~1(s,ξ2)(s−l)λ−2×(λ+ϕ~′(s)ϕ~(s)(s−l))2]ds>0,lim⁡ξ→∞sup⁡∫lξ[(ξ−s)λA(s)ϕ~(s)q~(s)−r~(s)ϕ~(s)4δ~1(s,ξ2)(ξ−s)λ−2×(λ−ϕ~′(s)ϕ~(s)(ξ−s))2]ds>0,

then (1) is oscillatory.

**Theorem 8**

Assume (8)–(10) hold, and

(45) $$\begin{array}{c} \int_{\xi_{0}}^{\infty }\left[A\left(s\right)\tilde{\phi }\left(s\right)\tilde{q}\left(s\right)-\frac{\tilde{r}\left(s\right){\left[{\tilde{\phi }}^{\prime }\left(s\right)\right]}^{2}}{4{\tilde{\delta }}_{1}\left(s,\xi_{2}\right)\tilde{\phi }\left(s\right)}\right]ds=\infty , \end{array}$$

where $\tilde{\phi }$ is defined as in Theorem 4. Then every solution of (1) is oscillatory or satisfies lim⁡_*t*→*∞*_ *x*(*t*) = 0.

**Proof**

Assume (1) has a nonoscillatory solution *x* on [*t*_0_, *∞*). Without loss of generality, we may assume *x*(*t*) > 0 on [*t*_1_, *∞*), where *t*_1_ is sufficiently large. By Lemma 1, we have *D*_*t*_^*α*^(*r*(*t*)*D*_*t*_^*α*^ *x*(*t*)) > 0, *t* ∈ [*t*_2_, *∞*), where *t*_2_ > *t*_1_ is sufficiently large, and either *D*_*t*_^*α*^ *x*(*t*) > 0 on [*t*_2_, *∞*) or lim⁡_*t*→*∞*_ *x*(*t*) = 0. Now we assume that *D*_*t*_^*α*^ *x*(*t*) > 0 on [*t*_2_, *∞*). Let *ω*(*t*), $\tilde{\omega }(\xi )$ be defined as in Theorem 4. Then we obtain (33), and furthermore,

(46) ω~′(ξ)≤−A(ξ)ϕ~(ξ)q~(ξ)−δ~1(ξ,ξ2)r~(ξ)ϕ~(ξ)ω~2(ξ)+ϕ~′(ξ)ϕ~(ξ)ω~(ξ)≤−A(ξ)ϕ~(ξ)q~(ξ)+r~(ξ)[ϕ~′(ξ)]24δ~1(ξ,ξ2)ϕ~(ξ),   ξ≥ξ2.

Substituting *ξ* with *s* in (46) and integrating (46) with respect to *s* from *ξ*_2_ to *ξ* yield

(47) ∫ξ2ξ[A(s)ϕ~(s)q~(s)−r~(s)[ϕ~′(s)]24δ~1(s,ξ2)ϕ~(s)]ds≤ω(ξ2)−ω(ξ)≤ω(ξ2)<∞,

which contradicts (45). So, the proof is complete.

**Theorem 9**

Assume (8)–(10) hold, and there exists a function *G* ∈ *C*([*ξ*_0_, *∞*), ℝ) such that *G*(*ξ*, *ξ*) = 0, for *ξ* ≥ *ξ*_0_, *G*(*ξ*, *s*) > 0, for *ξ* > *s* ≥ *ξ*_0_, and *G* has a nonpositive continuous partial derivative *G*_*s*_′(*ξ*, *s*). If

(48) lim⁡ξ→∞sup⁡1G(ξ,ξ0) ×{∫ξ0ξG(ξ,s){Kϕ~(s)q~(s)−ϕ~(s)φ~′(s)    +ϕ~(s)δ~1(s,ξ2)φ~1+1/γ(s)r~(s) −[(γ+1)φ~1/γ(s)ϕ~(s)δ~1(s,ξ2)+r~(s)ϕ~′(s)]γ+1(γ+1)γ+1[ϕ~(s)δ~1(s,ξ2)]γr~(s)}ds}=∞,

where $\tilde{\phi }$ is defined as in Theorem 4, then every solution of (1) is oscillatory or satisfies lim⁡_*t*→*∞*_ *x*(*t*) = 0.

**Proof**

Assume (1) has a nonoscillatory solution *x* on [*t*_0_, *∞*). Without loss of generality, we may assume *x*(*t*) > 0 on [*t*_1_, *∞*), where *t*_1_ is sufficiently large. By Lemma 1, we have *D*_*t*_^*α*^(*r*(*t*)*D*_*t*_^*α*^ *x*(*t*)) > 0, *t* ∈ [*t*_2_, *∞*), where *t*_2_ > *t*_1_ is sufficiently large, and either *D*_*t*_^*α*^ *x*(*t*) > 0 on [*t*_2_, *∞*) or lim⁡_*t*→*∞*_ *x*(*t*) = 0. Now we assume *D*_*t*_^*α*^ *x*(*t*) > 0 on [*t*_2_, *∞*). Let *ω*(*t*), $\tilde{\omega }(\xi )$ be defined as in Theorem 4. By (46), we have

(49) $$\begin{array}{c} A\left(\xi \right)\tilde{\phi }\left(\xi \right)\tilde{q}\left(\xi \right)-\frac{\tilde{r}\left(\xi \right){\left[{\tilde{\phi }}^{\prime }\left(\xi \right)\right]}^{2}}{4{\tilde{\delta }}_{1}\left(\xi ,\xi_{2}\right)\tilde{\phi }\left(\xi \right)}\leq {\tilde{\omega }}^{\prime }\left(\xi \right),\;\xi \geq \xi_{2}. \end{array}$$

Substituting *ξ* with *s* in (49), multiplying both sides by *G*(*ξ*, *s*), and then integrating both sides of (49) with respect to *s* from *ξ*_2_ to *ξ* yield

(50) ∫ξ2ξG(ξ,s){A(s)ϕ~(s)q~(s)−r~(s)[ϕ~′(s)]24δ~1(s,ξ2)ϕ~(s)}ds≤−∫ξ2ξG(ξ,s)ω~′(s)ds=G(ξ,ξ2)ω(ξ2)+∫ξ2ξHs′(ξ,s)ω(s)Δs≤G(ξ,ξ2)ω(ξ2)≤G(ξ,ξ0)ω(ξ2).

Then,

(51) ∫ξ0ξG(ξ,s){A(s)ϕ~(s)q~(s)−r~(s)[ϕ~′(s)]24δ~1(s,ξ2)ϕ~(s)}ds=∫ξ0ξ2G(ξ,s){A(s)ϕ~(s)q~(s)−r~(s)[ϕ~′(s)]24δ~1(s,ξ2)ϕ~(s)}ds+∫ξ2ξG(ξ,s){A(s)ϕ~(s)q~(s)−r~(s)[ϕ~′(s)]24δ~1(s,ξ2)ϕ~(s)}ds≤G(ξ,ξ0)ω~(ξ2)+G(ξ,ξ0)×∫ξ0ξ2|A(s)ϕ~(s)q~(s)−r~(s)[ϕ~′(s)]24δ~1(s,ξ2)ϕ~(s)|ds.

So,

(52) lim⁡ξ→∞sup⁡1G(ξ,ξ0){∫ξ0ξG(ξ,s){A(s)ϕ~(s)q~(s)−r~(s)[ϕ~′(s)]24δ~1(s,ξ2)ϕ~(s)}ds}≤ω~(ξ2)+∫ξ0ξ2|A(s)ϕ~(s)q~(s)−r~(s)[ϕ~′(s)]24δ~1(s,ξ2)ϕ~(s)|ds<∞,

which contradicts (48). So the proof is complete.

## 3. Applications of the Results

**Example**

Consider the following fractional differential equation:

(53) Dt1/3[t1/9Dt1/3Dt1/3x(t)]+t−1/3Dt1/3Dt1/3x(t)+t−2/3x(t)=0, t≥2.

In (1), if we set *t*_0_ = 2, *α* = 1/3, *a*(*t*) = *t* ^1/9^, *r*(*t*) ≡ 1, (*t*) = *t* ^−1/3^, *q*(*t*) = *t* ^−2/3^, then we obtain (53). So *ξ*_0_ = 2^1/3^/Γ(4/3), $\tilde{a}(\xi )=a(t)=t^{1/9}={\left[\Gamma (4/3)\xi \right]}^{1/3}$, $\tilde{r}(\xi )\equiv 1$, $\tilde{p}(\xi )={\left[\Gamma (4/3)\xi \right]}^{-1}$, $\tilde{q}(\xi )={\left[\Gamma (4/3)\xi \right]}^{-2}$. Furthermore, *A*(*ξ*) = exp⁡([Γ(4/3)]^−4/3^∫_*ξ*_0__^*ξ*^ *s* ^−4/3^ *ds*) = exp⁡(3[Γ(4/3)]^−4/3^[*ξ*_0_^−1/3^ − *ξ* ^−1/3^]), which implies 1 ≤ *A*(*ξ*) ≤ exp⁡(3[Γ(4/3)]^−4/3^ *ξ*_0_^−1/3^). On the other hand, ${\tilde{\delta }}_{1}(\xi ,\xi_{2})=\int_{\xi_{2}}^{\xi }(1/A(s)\tilde{a}(s))ds\geq {\left[\Gamma (4/3)\right]}^{-1/3}\exp\left(-3{\left[\Gamma (4/3)\right]}^{-4/3}\xi_{0}^{-1/3}\right)\int_{\xi_{2}}^{\xi }s^{-1/3}ds=\left(3/2\right){\left[\Gamma (4/3)\right]}^{-1/3}\exp\left(-3{\left[\Gamma (4/3)\right]}^{-(4/3)}\xi_{0}^{-1/3}\right)(\xi^{2/3}-\xi_{2}^{2/3})$, which implies $\lim_{\xi \to \infty }{\tilde{\delta }}_{1}(\xi ,\xi_{2})=\infty$, and then (8) holds. So, there exists a sufficiently large *T* > *ξ*_2_ such that ${\tilde{\delta }}_{1}(\xi ,\xi_{2})>{\left[\Gamma (4/3)\right]}^{2}$ on [*T*, *∞*). In (9),

(54) $$\begin{array}{c} \int_{t_{0}}^{\infty }\frac{\alpha t^{\alpha -1}}{\Gamma \left(1+\alpha \right)r\left(t\right)}dt=\int_{\xi_{0}}^{\infty }\frac{1}{\tilde{r}\left(s\right)}ds=\int_{\xi_{0}}^{\infty }ds=\infty . \end{array}$$

In (10),

(55) ∫ξ0∞1r~(ζ)∫ζ∞1A(τ)a~(τ)∫τ∞A(s)q~(s)ds dτ dζ≥[Γ(43)]−1/3exp⁡(−3[Γ(43)]−4/3ξ0−1/3)×∫ξ0∞∫ζ∞τ−1/3∫τ∞s−1ds dτ dζ=∞.

In (48), letting $\tilde{\phi }(\xi )=\xi$, we obtain

(56) ∫ξ0∞[A(s)ϕ~(s)q~(s)−r~(s)[ϕ~′(s)]24δ~1(s,ξ2)ϕ~(s)]ds=∫ξ0∞{A(s)[Γ(43)]−2−14δ~1(s,ξ2)}1sds=∫ξ0T{A(s)[Γ(43)]−2−14δ~1(s,ξ2)}1sds+∫T∞{A(s)[Γ(43)]−2−14δ~1(s,ξ2)}1sds≥∫ξ0T{A(s)[Γ(43)]−2−14δ~1(s,ξ2)}1sds+∫T∞34[Γ(43)]−21sds=∞.

Therefore, (53) is oscillatory by Theorem 8.

**Example 11**

Consider the following fractional differential equation:

(57) Dt1/2[t1/4Dt1/2Dt1/2x(t)]+Γ(3/2)tDt1/2Dt1/2x(t)+x(t)=0, t≥2.

In (1), if we set *t*_0_ = 2, *α* = 1/2, *a*(*t*) = *t* ^1/4^, *r*(*t*) ≡ 1, $p(t)=\Gamma (3/2)/\sqrt{t}$, *q*(*t*) ≡ 1, then we obtain (57). So *ξ*_0_ = 2^1/2^/Γ(3/2), $\tilde{a}(\xi )=a(t)=t^{1/4}=\sqrt{\Gamma (3/2)\xi }$, $\tilde{r}(\xi )\equiv 1$, $\tilde{p}(\xi )=\xi^{-1}$, $\tilde{q}(\xi )\equiv 1$. Furthermore, *A*(*ξ*) = exp⁡([Γ(3/2)]^−1/2^∫_*ξ*_0__^*ξ*^ *s* ^−3/2^ *ds*) = exp⁡([Γ(3/2)]^−1/2^[2*ξ*_0_^−1/2^ − 2*ξ* ^−1/2^]), which implies 1 ≤ *A*(*ξ*) ≤ exp⁡(2[Γ(3/2)]^−1/2^ *ξ*_0_^−1/2^). On the other hand, ${\tilde{\delta }}_{1}(\xi ,\xi_{2})=\int_{\xi_{2}}^{\xi }\left(1/A(s)\tilde{a}(s)\right)ds\geq {\left[\Gamma (3/2)\right]}^{-1/2}\exp\left(-2{\left[\Gamma (3/2)\right]}^{-1/2}\xi_{0}^{-1/2}\right)\int_{\xi_{2}}^{\xi }\left(1/\sqrt{s}\right)ds=2{\left[\Gamma (3/2)\right]}^{-1/2}\exp\left(-2{\left[\Gamma (3/2)\right]}^{-1/2}\xi_{0}^{-1/2}\right)(\sqrt{\xi }-\sqrt{\xi_{2}})$, which implies $\lim_{\xi \to \infty }{\tilde{\delta }}_{1}(\xi ,\xi_{2})=\infty$. So, there exists a sufficiently large *T* > *ξ*_2_ such that ${\tilde{\delta }}_{1}(\xi ,\xi_{2})>1$ on [*T*, *∞*).

From the analysis above, one can see the (8) holds. We now test (9) and (10). In (9),

(58) $$\begin{array}{c} \int_{t_{0}}^{\infty }\frac{\alpha t^{\alpha -1}}{\Gamma \left(1+\alpha \right)r\left(t\right)}dt=\int_{\xi_{0}}^{\infty }\frac{1}{\tilde{r}\left(s\right)}ds=\int_{\xi_{0}}^{\infty }ds=\infty . \end{array}$$

In (10),

(59) ∫ξ0∞1r~(ζ)∫ζ∞1A(τ)a~(τ)∫τ∞A(s)q~(s)ds dτ dζ≥[Γ(32)]−1/2exp⁡(−2[Γ(32)]−1/2ξ0−1/2)×∫ξ0∞∫ζ∞1τ∫τ∞ds dτ dζ=∞.

So, (9) and (10) hold. On the other hand, in (44), after putting $\tilde{\phi }(\xi )\equiv 1$, *λ* = 2, for any sufficiently large *l*, we have

(60) lim⁡ξ→∞sup⁡∫lξ[(s−l)λA(s)ϕ~(s)q~(s)−r~(s)ϕ~(s)4δ~1(s,ξ2)(s−l)λ−2(λ+ϕ~′(s)ϕ~(s)(s−l))2]ds≥lim⁡ξ→∞sup⁡∫lξ[(s−l)2s−1−1]ds=∞,lim⁡ξ→∞sup⁡∫lξ[(ξ−s)λA(s)ϕ~(s)q~(s)−r~(s)ϕ~(s)4δ~1(s,ξ2)(ξ−s)λ−2(λ−ϕ~′(s)ϕ~(s)(ξ−s))2]ds≥lim⁡ξ→∞sup⁡∫lξ[(ξ−s)2s−1−1]ds=∞.

So (44) holds, and then by Corollary 7 we deduce that (57) is oscillatory.
